# Upper circumpolar deep water influences microbial functional gene composition and diversity along the southern Central Indian Ridge and eastern Southwest Indian Ridge

**DOI:** 10.1128/spectrum.03306-23

**Published:** 2024-12-27

**Authors:** Sheryl Oliveira Fernandes, Dhiraj Paul, Surya Prakash Lankalapalli, Srinivas Rao Arvapalli, Vidya PJ, John Kurian Palayil

**Affiliations:** 1 National Center for Polar and Ocean Research, Ministry of Earth Sciences, Vasco-da-Gama, Goa, India; 2 National Center for Microbial Resource, National Center for Cell Science, Pune, India; Huazhong Agricultural University, Wuhan, China

**Keywords:** gene microarray, functional diversity, hydrothermal, microbial, Indian Ocean

## Abstract

Deep sea microbial communities play a significant role in global biogeochemical processes. However, the depth-wise metabolic potential of microbial communities in hydrothermally influenced Central Indian Ridge (CIR) and Southwest Indian Ridge (SWIR) remains elusive. In this study, a comprehensive functional microarray-based approach was used to understand factors influencing the metabolic potential of microbial communities and depth-driven differences in microbial functional gene composition in CIR and SWIR. Stratified water column sampling at surface, mid, turbid/plume layer, and near bottom was done along with pertinent environmental variables at various locations along the ridges. The majority of genes (~38%–41%) throughout the water column in both regions encoded for C-cycling, particularly starch degradation indicating the predominance of heterotrophy. Genes encoding for nitrate reduction and arsenic and mercury resistance were enriched in the turbid and/or near-bottom waters, suggesting a localized influence of hydrothermally derived substrates on the metabolic potential of microbial communities. Indices for microbial functional gene diversity (*H* = 9.18) and evenness (*J* = 0.90) were highest for samples from turbid waters at SWIR. Potential temperature-salinity profiles showed the presence of nutrient-rich upper circumpolar deep water (UCDW) at >2,000 m in the study areas. Principal component analysis revealed that inorganic nutrient availability largely influenced functional gene diversity in deeper waters. The study signifies that rather than hydrothermal input, nutrients brought into the region through the UCDW could have a larger impact on metabolic processes mediated by autochthonous microbial communities and consequently have implications on deep-sea productivity.

**IMPORTANCE:**

Little is known about depth-wise metabolic potential of microbial communities in hydrothermally influenced Central Indian Ridge (CIR) and Southwest Indian Ridge (SWIR) waters. In the present study, a comprehensive functional gene microarray approach was used to reveal the metabolic potential and depth-wise variation in microbial functional genes along the ridges. Up to 41% of microbial functional genes at both locations encoded for C-cycling. Availability of hydrothermally derived substrates in plumes detected along the ridges triggered an increase in the abundance of genes encoding for remediation of polycyclic aromatics, nitrate reduction, and arsenic and mercury resistance. Rather than hydrothermal input, the functional gene diversity at >2,000 m was largely influenced by inorganic nutrients transported by the nutrient-rich upper circumpolar deep water. Findings of this study are expanding the existing knowledge on new sites of hydrothermal activity along CIR and SWIR and gaining insights into ecosystem functioning in the deep sea.

## INTRODUCTION

Natural microbial communities can quickly respond to alterations in environmental variables in their habitat (1). In active hydrothermal chimneys, *in situ* temperature (2) and local fluid geochemistry (3) are important parameters shaping the structure and metabolic functions of microbial communities (4). Plume microbial communities have also been found to be discrete from those on the seafloor or in the sub-surface but have been suggested to retain some signatures of these habitats (5). The microbial community composition from vent plume to surrounding waters exhibits strong niche specificity, indicating some adaptation to hydrothermally derived reduced chemical entities (6). However, further away from vent sources, hydrothermal activity may have little influence on pelagic microbial communities, and it is largely controlled by water mass characteristics from each sample (7) or from particles sinking from the surface ocean.

Microbial community analysis from hydrothermal vents along Central Indian Ridge (CIR) and Southwest Indian Ridge (SWIR) has revealed epsilon-, gamma-, alpha-, and delta-proteobacteria along with members of phylum Bacteroidetes and Planctomycetes to be dominant lineages (2, 8). The archaeal community is dominated by Thaumarchaeota, Woesearchaeota, and Euryarchaeota (2, 9). Distinguished patterns of highly diversified bacterial and archaeal community structures along these ridges have been linked to regional geochemical conditions (2). Currently, limited information is available concerning functional partitioning of microbes in the water column. Moreover, investigations pertaining to environmental factors that shape microbial communities in hydrothermal plumes/background seawater, especially from the Central Indian Ocean are scarce. Both high-throughput sequencing and microarray technologies have been widely used to characterize microbial communities. However, these have distinct differences in sample preparation, data pre-processing, analysis, performance, and application (10). Though shotgun metagenomic sequencing has provided community-level information from a variety of environments, only a fraction of short-read sequences from complex communities may be useful for functional or phylogenetic analyses (10). Therefore, in this study, we used the GeoChip 5.0S microarray format (60 K × 8, 8 arrays with 60,000 probes each on one slide) to link microbial functional genes to ecosystem processes. This microarray is suitable for functionally profiling microbial communities since it is highly specific and sensitive (11). Microarray-based studies have been previously used to assess hydrothermal vent-associated microbial functional genes/populations (4). Information on depth-driven differences in microbial functional genes and their diversity in hydrothermally influenced waters is still unknown. It is important for ecosystem management/restoration strategies concerning the mining of seafloor polymetallic sulfides (PMS) in the future. We hypothesize that substrates derived through hydrothermal activity could alter microbial functional gene composition and diversity. They could favor proliferation of particular microbial groups and forms of metabolism which could in turn influence biogeochemical cycling in the aquatic system. We specifically aimed to investigate (i) depth-dependent alterations of microbial functional genes in the water column at CIR and SWIR, (ii) detect enrichment of biogeochemically important microbial metabolic pathways in turbid waters, and (iii) determine the influence of environmental factors on microbial functional gene diversity. Contrary to our hypothesis, results obtained in this study have revealed localized influence of hydrothermally derived substrates on altering microbial functional gene composition and diversity. However, it was observed that metabolic potential of microbes in deeper water along the ridges was largely governed by nutrient-rich upper circumpolar deep water (UCDW). These differences are important to gain a better understanding of hydro-biogeochemical processes in the region which are likely to be explored for PMS deposits in the near future.

## MATERIALS AND METHODS

### Sample collection and physico-chemical measurements

**Fig 1:**
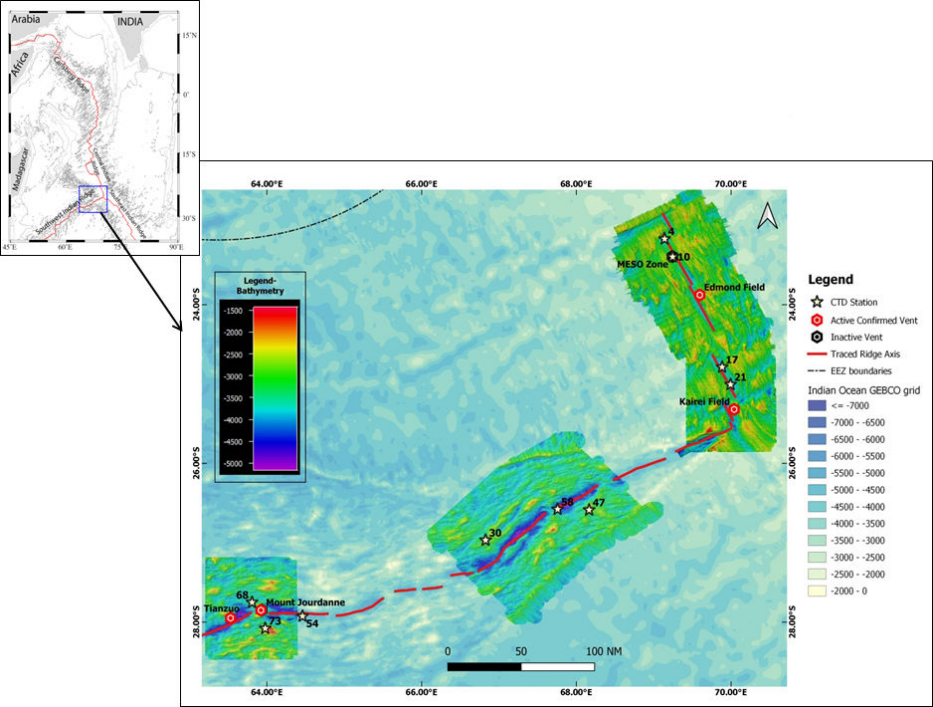
Multi-beam bathymetric map indicating sampling locations along CIR and SWIR. The blue box of the index map denotes the study area, while the red line denotes the Indian ridge system.

Stratified water column sampling was carried out during scientific expedition to CIR and SWIR (cruise nos. MGS 13 and 14) onboard *MGS Sagar* from January to March 2017. Sampling stations were selected along the ridge axis and flanks (Fig. 1). Hydrographic data and water samples were collected by the Sea Bird Electronics (SBE) 19plus SeaCAT profiler conductivity-temperature-depth (CTD) system. A total of 24 Niskin sampling bottles of 10 L capacity were mounted onto the CTD rosette. Vertical profiles of potential temperature (θ) and salinity (S) diagram (θ-S) at >2,000 m were used to identify water mass properties. Dissolved manganese (DMn) concentrations and turbidity anomalies were calculated (12). Sub-samples for measurement of pH were collected in 500 mL high-density polyethylene bottles, and pH was measured using a multi-parameter water quality meter (Eutech, model: CyberScan PCD 650). Similarly, sub-samples were analyzed within 4 h of sampling for dissolved oxygen (DO) and inorganic nutrients (ammonium, nitrite, nitrate, phosphate, and silicate) as described previously (13–17).

### Microbiological studies

Samples for microbiological analyses were collected in 10 L Jerricans that were initially washed with 70% ethanol and dried. Subsequently, before sample collection, they were thoroughly rinsed 3–4 times with seawater from the desired depth. Five liter of seawater was filtered through 0.2 µm filter (GTTP04700, Millipore, MA, United States), placed into 2 mL sterile storage vials, and immediately stored at −20°C until DNA extraction.

### Enumeration of total prokaryotic cells

The total prokaryotic cell (TPC) counts were carried out using epifluorescence microscopy (18, 19). Briefly, an aliquot of 5 mL seawater from each sampling depth was fixed using 250 µL of filter-sterilized buffered formalin (2% final concentration). Two milliliter of sub-sample was transferred to a sterile 5 mL self-standing vial that as wrapped in Al foil. A 20 µL volume of 4’, 6-diamidine-2’-phenylindole dihydrochloride working solution (final concentration = 300 µg/mL) was added to each sub-sample. The contents were mixed by pipetting and incubated in ice for 5 min. The samples were filtered through a *Millipore* 0.2 µm black Isopore polycarbonate filter paper followed by washing with 10–15 mL of sterile phosphate-buffered saline. The black polycarbonate filter was then mounted onto a slide using immersion oil and covered with a cover slip. Microbial cells were counted in 15 random microscopic fields to obtain a reliable mean using Leica epifluorescence microscope (model: DM6B) and expressed as cells per liter.

### Analysis of microbial functional genes

Community DNA was extracted from 30 samples using PowerWater DNA Isolation Kit (Mo Bio Laboratories, CA, USA) according to the manufacturer’s protocol. The DNA quality was evaluated by absorbance ratios at *A*_260_/*A*_280_ using a NanoDrop ND-1000 spectrophotometer (NanoDrop Technologies Inc., Wilmington, DE). The extracted DNA was stored at –20°C for use as templates for PCR amplifications and GeoChip 5.0S microarray analysis. Detailed description of DNA amplification, labeling, GeoChip hybridization, data pre-processing, analysis of detected functional genes, and statistical tests used has been provided in the supplementary section.

### Amplification of DNA, labeling, and GeoChip hybridization

Aliquots of community DNA were amplified using the TempliPhi kit (GE Healthcare Life Sciences, USA) with a modified buffer containing spermidine (0.1 mM) and a single-stranded binding protein (267 ng mL^−1^) to improve the amplification efficiency (20). Samples were amplified for 6 h.

Amplified DNA (~2 µg) was mixed with 5.5 µL random primers (Life Technologies, random hexamers, 3 µg/µL), made up to a final volume of 35 µL with nuclease-free water, heated to 99°C for 5 min, and immediately placed on ice. A 15 µL labeling master mix {(2.5 µL of dNTP [5 mM dAGC-TP and 2.5 mM dTTP], 0.5 µL of Cy-3dUTP [25 nM; GE Healthcare], 1 µL of Klenow [Imer Inc., CA, USA; 40 U mL^−1^], 5 µL Klenow buffer, and 2.5 µL of water)} was added. The samples were incubated at 37°C for 6 h in a thermocycler and thereafter at 95°C for 3 min to inactivate the enzyme. After the addition of Cy3, samples were protected from light as much as possible. Labeled DNA was cleaned using a QIAquick PCR purification kit (Qiagen, Hilden, Germany) as per the manufacturer’s instructions and then dried down at 45°C for 45 min in a *Savant* SpeedVac (Thermo Fisher Scientific Inc., Asheville, NC).

Labeled DNA was rehydrated with 27.5 µL de-ionized water. Hybridization solution was added that contained 99.4 µL: 63.5 µL 2× Hi-RPM hybridization buffer (Agilent, CA, USA), 12.7 µL pre-prepared 10× blocking agent (Agilent), 12.7 µL formamide, and 5.5 µL Cot-1 DNA (common oligo reference standard [CORS]) (21). The contents were mixed well and spun to collect liquid at the bottom of the tube. The tubes were incubated at 95°C for 5 min and then at 37°C for 30 min. Gasket slides were placed into a SureHyb chamber, and 120 µL of the samples was loaded onto the gasket slide. The microarray was placed on top, i.e., array side down, and the chamber hand was tightened to create a seal. The chamber was placed in a rotator/incubator. Samples were hybridized on a GeoChip 5.0S microarray chip for 20–22 h at 67°C in an Agilent microarray hybridization oven (Agilent Technologies, CA, USA).

After hybridization, slides were disassembled at room temperature and incubated for 5 min. The slides were washed using the Oligo aCGH/ChIP-on-Chip Wash Buffer kit (Agilent). GeoChips were imaged using the NimbleGen MS 200 microarray scanner (Roche NimbleGen, Madison, WI, USA) as a Multi-TIFF. The data were extracted using the Agilent Feature Extraction program.

### Data pre-processing and analysis of detected functional genes

Data normalization and quality filtering were performed with multiple steps (21). First, the average signal intensity of the CORS which is used for data normalization and comparison was calculated for each array. The maximum average value was applied to normalize the signal intensity of samples in each array. Thereafter, the sum of the signal intensity of samples was calculated for each array. The maximum sum value was applied to normalize the signal intensity of all spots in an array. This produced a normalized value for each spot in each array. Spots were then scored as positive and retained if the signal-to-noise ratio (SNR = [signal mean – background mean]/background SD) was ≥2.0, and the coefficient of variation of the background was <0.8. Additionally, spots with signal intensity <200 were discarded. Spots that were detected in <2 samples either within a replicate group or across all samples were removed. All raw and processed GeoChip 5.0S data were deposited to NCBI Gene Expression Omnibus (GEO, https://www.ncbi.nlm.nih.gov/geo/) under the accession number 22156631.

The pre-processed GeoChip data were used for enumerating relative abundance of functional gene categories/sub-categories. This was done by calculating the sum of detected probe numbers in each functional gene category in one sample and dividing by the total detected probe number in the same sample.

### Statistical analyses

Statistical differences in the mean proportion of genes in each functional category/subcategory among samples were analyzed by one-way analysis of variance (ANOVA) followed by Duncan’s Multiple Range test. A significance level of *P* < 0.05 was adopted for all comparisons with a 95% confidence interval. Diversity indices, viz., Shannon–Weaver index (H′), Simpson index (1-D), Inverse Simpson (1/D), and Pielou’s evenness (J), were used to calculate the functional gene diversity. Mean values of abiotic/biotic variables and diversity indices were compared by one-way ANOVA at a significance level of 5% (*P* < 0.05) for CIR and SWIR samples. Associations between diversity indices/proportion of genes and biogeochemical variables were calculated by Pearson’s correlation to identify possible linkages. Principal component analysis (PCA) was performed to understand the influence of environmental variables on functional gene diversity. To identify the interrelations among the samples based on bacterial functional diversity distribution, a UPGMA (unweighted pair group method with arithmetic mean)-based clustering analysis was performed. The MVSP 3.1 software was used for UPGMA dendrogram analysis (22). Most of the statistical analyses were performed using the R program.

## RESULTS

### Detection of turbidity anomalies and water masses

**Fig 2:**
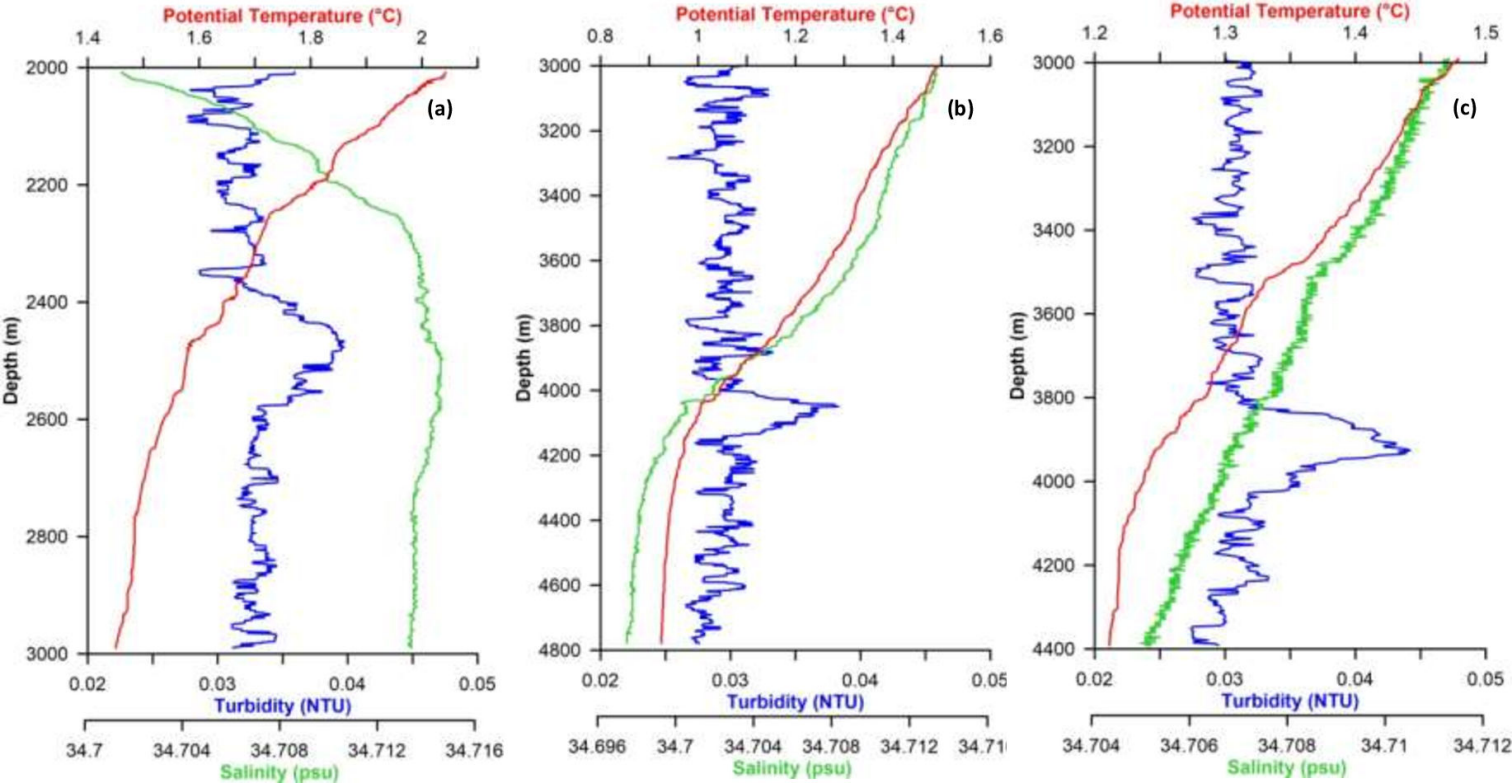
Vertical profiles of potential temperature, salinity, and turbidity at (**a**) CTD 17, (**b**) CTD 54, and (**c**) CTD 58.

Vertical profiles of optical backscatter clearly showed the presence of turbid layers in the deep waters (Fig. 2). Turbidity anomaly varies from 0.01 to 0.018 dNTU for turbid layers in CIR and SWIR with thickness of ~200 m. At CTD 17, an anomaly of 0.01 dNTU was observed between 2,400 and 2,600 m (Fig. 2a). At SWIR, turbidity maxima were observed between 4,000 and 4,180 m at CTD 54 (Fig. 2b) and 3,800–4,000 m for CTD 58 (Fig. 2c).

**Fig 3:**
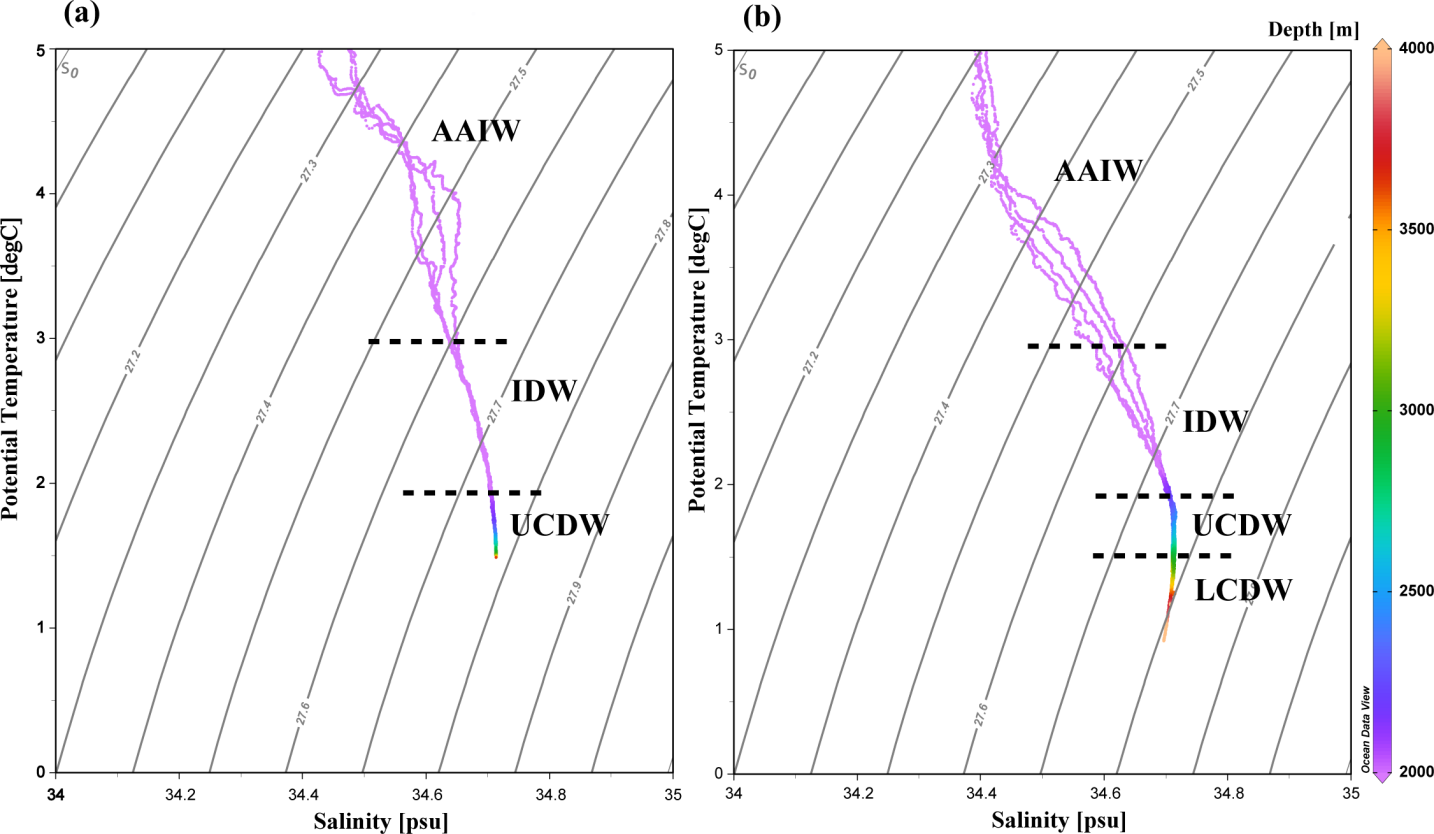
Potential temperature – salinity diagram (θ-*S*) plots for (**a**) CIR and (**b**) SWIR at ≥2,000 m. AAIW, Antarctic Intermediate Water; IDW, Indian Deep Water.

Potential temperature (θ) and salinity (S) diagram (θ-*S*) at CIR (Fig. 3a) and SWIR (Fig. 3b) showed the presence of three water masses in the regions, viz., Antarctic Intermediate Water (θ = 2.9°C–5°C, *S* = 34.4–34.6) (23), Indian Deep Water (θ = 0.6°C–3.5°C, *S* = 34.5–34.7) (23), and Circumpolar Deep Water. The Antarctic Intermediate Water and Indian Deep Water were found from 1,000 to 2,000 m. The Circumpolar Deep Water mass is further separated into UCDW (θ = 1.6°C–1.9°C, *S* = 34.6–34.7) (24) and lower circumpolar deep water (LCDW, θ = 1.25°C–1.5°C, *S* = 34.7) (24, 25). The UCDW is characterized by lower DO and high inorganic nutrient content and was detected between 2,000 and 3,800 m in both regions. The LCDW has relatively higher oxygen and salinities (26) and was detected only at SWIR. Schematic representation of ocean circulation and associated path of water masses at >2,000 m in the southwestern Indian Ocean have been described earlier (27).

### Environmental characteristics

Variation of abiotic and biotic parameters in the water column along CIR and SWIR have been shown in Table 1. Near-bottom and surface temperature (°C) of seawater along CIR was 1.72 and 26.98 and SWIR was 1.33 and 27.72. Salinity varied with depth and was higher at the surface at CIR (ANOVA, *F* = 187.02, *P* < 0.05) and SWIR (*F* = 76.85, *P* < 0.05). At CIR, DO content in surficial and near-bottom waters was ~4 mL L^−1^ unlike at SWIR where its concentration in near-bottom waters was relatively lower. Significantly low DO values (3.06–3.90 mL L^−1^) were recorded mid-depth at all sampling locations along CIR (*F* = 47.17, *P* < 0.00002) and SWIR (*F* = 16.95; *P* < 0.00009). The deeper waters at CIR showed high nitrate content of up to 38.25 µmol L^−1^ and phosphate at 2.90 µmol L^−1^. Silicate concentration also varied significantly with depth varying from 0.59 to 104.36 µmol L^−1^ at CIR (*F* = 244.83; *P* < 1.425e-8) and 1.10–119.70 µmol L^−1^ at SWIR (*F* = 11.01; *P* < 0.0007). Concentration of DMn varied with depth with relatively higher concentration in the deeper waters, i.e., >2,400 m at both CIR (*F* = 14.99; *P* < 0.001) and SWIR (*F* = 30.48; *P* < 0.000004). An elevated DMn concentration (2–4 times higher as compared to background) was detected in turbid layers, i.e., 2,450 m at CTD 17 along CIR and 3,920 m at CTD 58 along SWIR. High concentrations of DMn in CTD 17 are attributed to the recently reported new ultramafic-hosted hydrothermal field (12), where the measured DMn concentrations were up to 112 nM.

**TABLE 1:** Variation of physico-chemical and microbiological parameters at CIR and SWIR^*a*^

Location	Station no.	Depth	Temp	Salinity	pH	DO	Chl a	Ammonium	Nitrite	Nitrate	Phosphate	Silicate	DMn	TPC
		(m)	(°C)			(mL L^−1^)	(mg m^3^)	(µmol L^−1^)	(µmol L^−1^)	(µmol L^−1^)	(µmol L^−1^)	(µmol L^−1^)	(nmol L^−1^)	(log nos. L^−1^)
CIR	4	10	26.55	35.06	8.04	4.75	0.03	0.30	0.02	0.10	0.02	0.93	1.77	8.66
		1,800	2.70	34.68	7.41	3.63	0.03	0.29	0.04	34.50	2.40	86.18	0.73	8.06
		3,597	1.79	34.71	7.61	4.02	0.04	0.28	0.04	38.25	2.48	104.36	2.56	8.11
	10	10	26.77	35.03	8.03	4.74	0.03	0.35	0.02	0.00	0.05	1.86	2.91	8.08
		1,500	3.39	34.62	7.47	3.28	0.03	0.38	0.02	38.60	2.90	67.01	0.81	8.42
		2,800	1.78	34.71	7.65	4.03	0.04	0.36	0.02	34.20	2.88	92.38	3.77	8.70
	17	10	26.98	35.04	8.09	4.74	0.04	0.24	0.04	0.05	0.00	0.59	1.45	8.45
		1,365	3.67	34.59	7.53	3.24	0.03	0.28	0.04	33.34	2.88	68.21	0.21	8.69
		2,450	1.80	34.71	7.59	4.01	0.04	0.24	0.04	32.08	2.52	89.15	5.42	8.28
		3,050	1.76	34.71	7.53	4.04	0.02	0.33	0.04	31.44	2.48	92.35	3.18	8.42
	21	10	26.89	35.05	8.10	4.73	0.04	0.24	0.07	0.00	0.00	1.5	1.87	8.33
		1,930	2.21	34.70	7.50	3.90	0.03	0.32	0.07	35.40	2.60	79.6	1.27	8.57
		3,865	1.72	34.71	7.57	3.97	0.02	0.36	0.07	32.60	2.67	101.8	2.38	8.16
SWIR	30	10	27.32	35.46	8.03	4.16	0.04	0.13	0.09	0.10	0.07	1.1	1.32	8.68
		1,650	2.96	34.65	7.61	3.03	0.04	0.17	0.09	35.80	2.55	89.3	1.22	8.54
		3,307	1.72	34.71	7.67	3.51	0.03	2.05	0.09	35.20	2.81	115.6	2.11	8.58
	47	10	27.72	35.46	8.08	4.14	0.05	0.47	0.04	0.10	0.02	1.8	1.58	8.94
		1,425	3.27	34.57	7.61	3.06	0.03	0.31	0.15	29.40	2.60	77.2	0.56	8.56
		2,850	1.72	34.71	7.71	3.56	0.03	0.51	0.04	28.70	2.38	105.9	2.87	8.66
	54	4,055	1.33	34.70	7.68	3.77	0.05	0.18	0.02	25.64	2.41	39.85	ND	8.75
	58	10	26.52	35.53	8.1	4.27	0.02	0.32	0.04	0.20	0.07	5.19	1.60	8.58
		2,195	2.02	34.71	7.65	3.44	0.03	0.28	0.06	34.48	2.50	35.58	0.85	8.87
		3,920	1.57	34.71	7.7	3.61	0.03	0.30	0.11	31.60	2.45	38.33	5.38	8.06
		4,390	1.58	34.71	7.67	3.63	0.02	0.62	0.06	32.87	2.45	37.90	1.82	8.87
	68	10	26.65	35.81	8.1	4.20	0.06	0.62	0.02	0.50	0.12	1.2	0.93	8.70
		1,900	2.29	34.69	7.59	3.43	0.03	0.22	0.07	29.10	2.60	66.9	0.32	8.65
		3,980	1.50	34.71	7.61	3.67	0.02	0.58	0.07	36.0	2.52	119.7	1.43	8.57
	73	10	26.31	35.78	8.07	4.20	0.04	1.15	0.04	0.20	0.40	3.24	1.20	8.51
		1,000	5.26	34.40	7.57	3.84	0.01	0.23	0.08	31.96	2.24	33.89	0.38	8.75
		2,165	2.09	34.70	7.61	3.46	0.04	0.26	0.13	27.29	2.48	63.80	0.21	8.51

^*a*^ Samples from only turbid layer were collected at CTD 54 where hydrothermal activity has previously been reported by German et al., 1998. NA- Not analyzed.

### Functional gene composition and diversity

**Fig 4:**
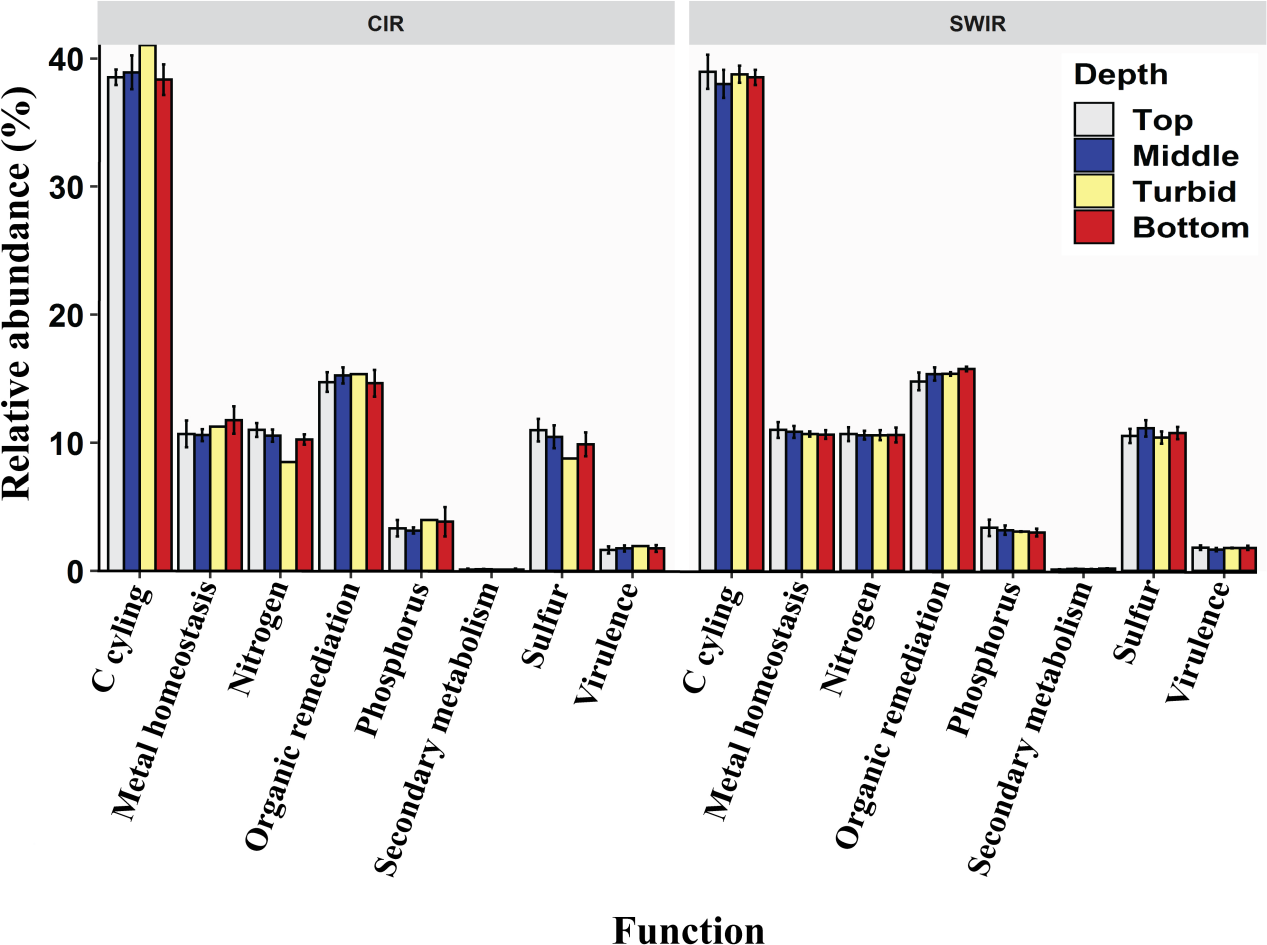
Proportion of genes in major functional gene categories. The percentage of genes was calculated by dividing the total signal intensity of genes from each major functional gene category by the total intensity of all genes detected for each sample. To represent the relative abundance of genes depth-wise, values from each depth, viz., top, middle, turbid, and near bottom, were pooled and averaged.

Microbes from CIR and SWIR were primarily involved in C-cycling (38%–41%), whereas a considerable fraction represented those involved in remediation of organic compounds (15%), transformation of metals (~12%), sulfur (9%–11%), and nitrogen (8%–11%) (Fig. 4). All through the water column, no significant differences in proportion of genes recovered from the two geographically different locations could be observed for major gene categories (Fig. 4). In this manuscript, the aforementioned dominant metabolisms are dealt with in more detail in order to understand functional processes mediated by microbial communities in hydrothermally influenced waters.

Alpha diversity was used to analyze the diversity of genes within the community (Table 2). No significant depth-wise variations in microbial functional gene diversity indices *H* and 1/(1 *– D*) were observed. Though evenness was comparable for all samples (Table 2), Shannon–Weaver index revealed greater functional gene diversity at SWIR than at CIR (Table 2). Inverse Simpson’s index was highest for sample collected at CTD 68, 3,980 m followed by those from the turbid layers at SWIR.

**TABLE 2:** Diversity indices based on all probes detected for CIR and SWIR samples

Location	Station no.	Depth	Shannon (*H*)	Simpson (*D*)	Invsimpson (1/1 *– D*)	Pielou’s evenness (*J*)
CIR	4	10	8.337	0.999	1432.210	0.850
	4	1,800	9.042	1.000	3080.193	0.892
	4	3,597	9.195	1.000	3688.199	0.888
	10	10	7.797	0.998	606.778	0.837
	10	1,500	8.939	1.000	2399.205	0.884
	10	2,800	9.032	1.000	3231.626	0.890
	17	10	8.681	0.999	1162.571	0.862
	17	1,365	8.808	1.000	2101.279	0.877
	17	2,450	8.419	0.999	1299.836	0.863
	17	3,050	8.731	1.000	2438.342	0.869
	21	10	8.543	0.999	1188.147	0.878
	21	1,930	8.660	0.999	1742.249	0.871
	21	3,865	8.836	1.000	2581.260	0.878
						
SWIR	30	10	9.018	1.000	2734.688	0.887
	30	1,650	9.068	1.000	3122.462	0.897
	30	3,307	8.879	1.000	2894.259	0.881
	47	10	8.996	1.000	2847.227	0.885
	47	1,425	9.047	1.000	3446.265	0.885
	47	2,850	8.375	0.999	1729.480	0.840
	54	4,055	9.235	1.000	4022.256	0.906
	58	10	8.820	1.000	2388.020	0.869
	58	2,195	8.811	1.000	3683.893	0.868
	58	3,920	9.117	1.000	4064.102	0.896
	58	4,390	9.059	1.000	2979.375	0.892
	68	10	8.979	1.000	3794.828	0.883
	68	1,900	8.910	1.000	2629.140	0.878
	68	3,980	9.266	1.000	5610.121	0.907
	73	10	8.492	0.999	1165.501	0.880
	73	1,000	8.877	1.000	2288.070	0.883
	73	2,165	9.069	1.000	3224.908	0.891

### Carbon cycling

Genes mediating C-fixation, C-degradation, and methane metabolism were detected in all samples. Autotrophic carbon fixation in ridge waters was dominated by genes involved in the Calvin cycle (Fig. S1). At CIR, these genes were predominant in the turbid layer (4.4%) as compared to SWIR where their abundance peaked at the surface (4.36%; Fig. S1a). Genes mediating CO_2_ fixation via alternate pathways, viz., the reductive tricarboxylic acid (rTCA) cycle and reductive Acetyl-CoA (acetyl coenzyme A) pathway, were also detected but to a lesser extent, i.e., <0.4% of the total genes. At CIR, genes mediating rTCA cycle were also found to be marginally higher (0.37%) in the turbid layer as compared to other depths.

Genes mediating degradation of about 24 different carbon compounds were detected (Fig. S1b). In both regions, those involved in starch metabolism were the most abundant followed by those for chitin and hemicellulose degradation. In the turbid layer above CIR, large portion of genes was involved in the degradation of starch (13.11%) and chitin (6.24%) unlike at the turbid layer above SWIR where chitin (5.52%) and hemicellulose (4.29%) degrading genes were more abundant.

Genes responsible for C1 compound metabolisms were detected in both CIR and SWIR sampling sites. Process for methane formation, i.e., methanogenesis (0.68%–0.83%), exceeded those for methane oxidation (CH_4_ to CO_2_ formation; 0.32%–0.49%) at both CIR and SWIR (Fig. S1c). Interestingly, genes for methanogenesis showed higher proportion in the turbid layer at SWIR compared with other deeper depths (Fig. S1c).

### Metabolism of organic compounds

Among various genes involved in degradation of organic compounds, a noticeable increase in proportion of genes for degradation of polycyclic aromatics was noticed for sample from the turbid layer at CIR as compared to other depths (Fig. S2). Genes for degradation of aromatic carboxylic acid, BTEX (group of contaminants consists of benzene, ethylbenzene, toluene, and three isomers of xylene), related aromatics, chlorinated aromatics, and chlorinated solvents were the other dominant types, making up >1% of the total relative gene abundance.

### Nitrogen, sulfur, and phosphorus cycling

Among various genes mediating N-cycle, our microarray data set provided genetic evidence for the predominance of denitrification in ridge waters. Genes mediating nitrate, nitrite, and nitrous oxide reduction contributed 3%–6% of the total genes (Fig. S3) at both CIR and SWIR. Genes mediating nitrogen fixation were also observed at all depths contributing 1.87%–2.94% of the total genes. At CIR, genes for ammonification were higher in proportion in the mid and near-bottom waters (~2.6%), whereas at SWIR, they were highest in the turbid layer (2.7%). Among the genes mediating sulfur cycling, those responsible for sulfite reduction (3.4%–4.7%) and sulfur oxidation (2.2%–2.6%) dominated throughout the water column (Fig. S4). Though lower in abundance (<0.8%), genes encoding catalytic subunit of adenylylsulfate reductase which reversibly catalyzes the reduction of adenosine 5'-phosphosulfate to sulfite and AMP during dissimilatory sulfate reduction were also detected in ridge waters. Genes mediating dimethylsulfoniopropionate (DMSP) degradation were also detected at all depths in both regions. Their abundance particularly in the turbid layer at SWIR was marginally higher in comparison to the other depths sampled.

Phosphorus cycling in ridge waters was dominated by an abundance of genes encoding for hydrolysis of polyphosphate (polyP) that leads to liberation of inorganic phosphate (Fig. S5). At CIR, abundance of these genes was relatively higher in the deeper waters (turbid and near bottom), whereas at SWIR, they were higher at the surface. Genes for polyphosphate synthesis and phytic acid hydrolysis were <0.4% at all sampled depths in both regions.

### Metal resistance

The microarray data set revealed high abundances for genes resistant to arsenic (4.4%–5.0%), tellurium, and mercury (2.3%–3.5%; Fig. S6). Relative abundance of genes for mercury was particularly higher in the turbid layer at CIR. Genes for copper, chromium, and those encoding for the enzyme manganese peroxidase which oxidizes Mn^2+^ to Mn^3+^ were also detected all through the water column in both regions with no significant depth-wise changes in the relative abundance.

### Interrelationship between microbial functional gene diversity and environmental parameters

Interrelationships between abiotic/biotic variables and diversity indices were identified by Pearson’s correlation analysis. At CIR, nutrients, viz., silicate, nitrate, and phosphate, were positively correlated with depth (Fig. S7). Interestingly, these abiotic parameters also exerted a strong positive influence on microbial functional gene diversity. A negative relationship of diversity indices with DO, temperature, and salinity was observed. At SWIR, the enrichment of nitrate, phosphate, and silicate in deeper waters was strengthened through a positive correlation of these nutrients with depth. However, ammonium content in the water column showed an inverse relationship with depth, whereas diversity indices were positively correlated (Fig. S8).

**Fig 5:**
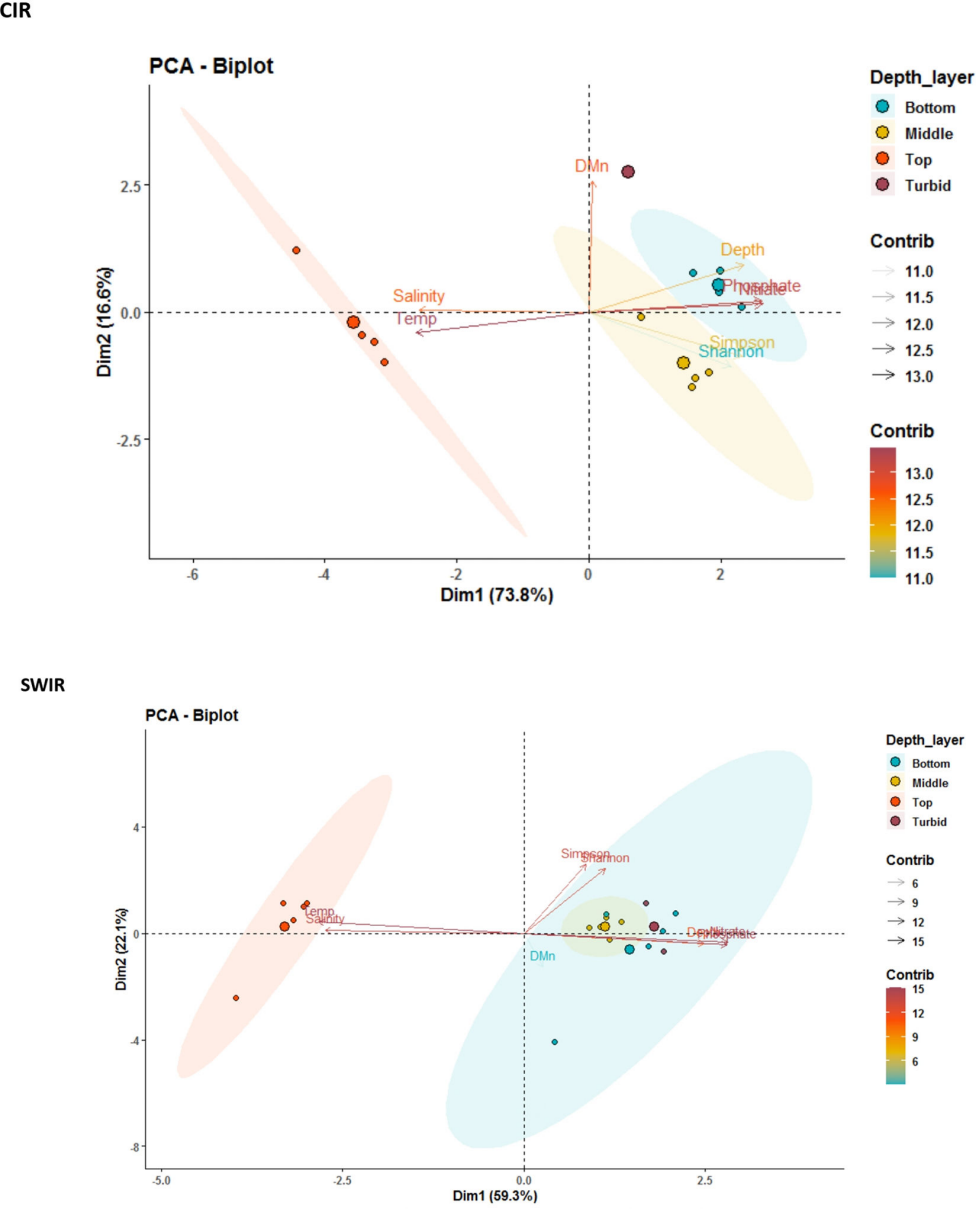
PCA of diversity indices and environmental parameters for (**a**) CIR and (**b**) SWIR samples grouped based on depth (surface/top, middle, turbid, and bottom). Chl. a, chlorophyll a; pH, hydrogen ion concentration. Eigenvectors (projected parameters) point in the direction of maximum variation and the length of each parameter is equivalent to their rate of change in the data set. Eigenvectors in opposite directions imply negative correlations while those in the same direction are positively correlated. Oblique angles between two vectors imply a certain degree of relatedness between vectors, whereas right angles between vectors show that they are independent from one another (28).

A PCA biplot was used to analyze the extent of variation in environmental parameters and bring out strong distribution patterns (Fig. 5). Only the first two axes were taken into account which comprised 90.4% (Fig. 5a) and 81.4% (Fig. 5b) of data’s total variability at CIR and SWIR, respectively. The PCA biplot of CIR data showed that sampling sites formed three separate clusters. Cluster I consisted of samples from near bottom. Cluster II included samples from the mid-water column, while Cluster III comprised only surface samples. Phosphate, depth, and diversity indices were negatively related to axis 1 and characterized groups I and II of cluster analysis. Samples from the surface were positively characterized to axis 1 by higher temperature, DO, and salinity. The PCA biplot of SWIR data also revealed clustering of samples from different strata of the water column. However, an overlap of clusters formed by aggregation of samples from the mid and near-bottom regions was observed. Phosphate and nitrate content showed very strong positive correlation with depth in the region. A positive correlation with functional diversity indices was also observed. As in CIR, the microbial functional gene diversity in surficial water was seen to be largely controlled by variations in temperature and salinity. In deeper waters, nutrient availability was more important in influencing the functional diversity of microbial communities.

## DISCUSSION

This study along CIR and SWIR provides deep insights on prevailing environmental conditions, metabolic pathways of microbial communities, and the links between microbial functional gene diversity and environmental variables. Physical observations revealed the presence of four prominent water masses in the deeper water column along CIR and SWIR (Fig. 3). Of these, the colder and saltier LCDW was observed only at >3,000 m in SWIR, indicating that complex topography of the ridge could be preventing free movement of the water mass toward CIR. A number of transform faults, such as Gallieni, Atlantis II, Melville, Gazelle, Gauss, and Novara between 52 °S and 61°S along SWIR (29), could allow intrusion of abyssal waters from the Weddell basin northward through the relatively unobstructed Crozet Basin (30). The UCDW which is characterized by oxygen depletion and an enhancement of nitrate and phosphate content (31) was detected in both regions. Stratified water column sampling employed in this study revealed relatively lower oxygen content between 2,000 and 3,000 m. Both physical signatures (Fig. 3) and elevated nitrate, phosphate, and/or silicate content with depth at >2,000–3,000 m (Fig. S7 and S8) further supported the presence of nutrient-rich UCDW at CIR and SWIR. In the Southern Ocean, contact with sediments increases the silica content of the bottom water (30). Furthermore, utilization of nitrate and phosphate by phytoplankton is largely incomplete due to light (32) and iron (33) limitations. Though nitrate δ^15^N is relatively invariant (~4.8‰ ± 0.2‰) below 2.5 km in LCDW, isotopic composition of the nutrient in UCDW is ~0.7‰ greater than that of LCDW. This has been attributed to denitrification in water masses in the low latitude with which the UCDW communicates (34). Low-nutrient content in surficial waters could be attributed to enhanced biological productivity in the euphotic zone (Table 1).

Live continuous measurements of hydrothermal plume signatures employed during our expeditions revealed prominent turbidity signatures at one location along CIR and two locations along SWIR (Fig. 2). Turbidity anomalies can be detected a few kilometers away from an active source. However, fine-grained suspended plume particles in hydrothermal plumes are quasi-conservative over many days (35). Hence, we used DMn as an additional chemical tracer for plume exploration studies. Dissolved Mn (II) concentrations can range from 5 mM within high-temperature vent fluids to just above ambient seawater concentration in distant plumes (36). In our study, concentration of DMn in turbid waters was ~2–4× higher (Table 1) compared to near-bottom waters supporting hydrothermal source of the turbid layers in this region.

Hydrothermal vent fluids and surrounding water can greatly influence the metabolic potential of microbial communities. Genome-based metabolic inferences have indicated high metabolic diversity of microbial assemblages from hydrothermal environments (37). The GeoChip 5.0 microarray identifies most common functional genes found in nature allowing biogeochemical pathways to be detected. Our findings showed that the majority of the functional genes targeted by the microarray were present at both ridges and throughout the water column at CIR and SWIR. They were involved in C-cycling, particularly C-degradation (Fig. 4; Fig. S1b). Most of the C-degradation genes in both regions were encoded for starch metabolism (Fig. 1b). Starch and starch-containing substrates are widespread in nature. The polymeric carbohydrate is easily degraded to α-D-glucose using exoenzymes (38). Bacterioplankton at CIR and SWIR appears to prefer labile forms of carbon like starch which could serve as sources of energy and carbon. Thus, they could be playing a key role in the marine C-cycle. Microbial degradation of organic compounds also causes loss of structural carbohydrates like cellulose and hemicelluloses which are more recalcitrant than starch. Up to 4% of functional genes in the water column at CIR and SWIR were encoded for metabolisms of these compounds. Chitin serves as a structural component in the exoskeletons of fungi and invertebrates. Chitinolytic bacteria are principal degraders of chitin (39). Marine organisms use it as an energetic source of nitrogen and carbon (40). Up to 6% of genes for C-degradation at CIR and SWIR were for chitin degradation with a maximum in the turbid waters. Known active hydrothermal vent fields, viz., Edmond, Kairei, Yokoniwa, Tiancheng, and Tianzuo, are located in the vicinity of our study areas. A variety of vent-associated fauna have been reported at these sites (41–43). These include major chitin contributors, viz., actinians, polycheates, gastropods, bivalves, cirripedes, shrimps, brachyuran crabs, galatheids, holothurians, and fishes (44). Elevated chitin-degrading genes in hydrothermal plumes are thus suggestive of input of the biopolymer from faunal communities along hydrothermal vents at CIR and SWIR.

Microbial chemosynthesis primarily supports the deep-sea hydrothermal systems (44, 45). Chemosynthetic primary production at deep-sea hydrothermal vents globally has a potential of ~10^13^ g biomass y^−1^, which comprises nearly 0.02% of the worldwide photosynthesis-based primary production in the oceans (46). Food webs in these systems rely on petroleum-based organic matter and thiotrophy via the Calvin–Benson–Bassham (CBB) and rTCA cycles (47). However, the extent of methanotrophy vs autotrophy based on CBB or rTCA cycles has been found to vary among and within vent fields depending upon the concentration of CH_4_ and H_2_S (48). We found Calvin cycle to be the most widespread at CIR and SWIR, indicating that aerobic chemolithoautotrophs have a preference for using the oxygen-tolerant, but energy-demanding, pathway. This study also showed that genes involved in Calvin cycle (Fig. S1a) were highest in the turbid layer at CIR where they could efficiently aid in C-fixation. This observation also strengthens earlier findings that Gammaproteobacteria have a preference to occupy habitats characterized by lower temperature and are away from active vent source (5, 49, 50). A majority of deep-sea vent chemoautotrophs can also fix inorganic carbon via the rTCA cycle (51). The CBB pathway and rTCA have been identified in sulfur-oxidizing autotrophic bacteria from hydrothermal chimneys on SWIR (8). This pathway seems restricted to bacteria and operates in Aquificales, Chlorobiales, Nitrospirae, Epsilonproteobacteria, and single strains of other proteobacterial groups (52). At CIR and SWIR, members belongings to the aforementioned classes constitute a minor fraction of the microbial community. Therefore, an overall lower contribution of rTCA cycle in C-fixation could be expected (Fig. S1a).

The present study shows that genes for methanogenesis were ~2× higher than those for methane oxidation in the turbid layer at SWIR (Fig. S1c). In hydrothermal ecosystems, lithoautotrophic methanogenesis is sustained due to hydrogenotrophic, hyperthermophilic methanogens which utilize H_2_ to reduce CO_2_ (53) via the reductive acetyl-CoA pathway (54). Both H_2_ and CH_4_ are formed by serpentinization (55). Fluids from active vents along CIR and SWIR especially those from the Kairei hydrothermal field (KHF) are considerably enriched with H_2_ and have a remarkably low CH_4_/H_2_ ratio (42, 56–58). Significantly low ^δ^D(H_2_) and δ^13^C(CH_4_) levels in KHF vent fluids are indicative of the occurrence of microbial methanogenesis (53). A greater number of genes for methanogenesis in the water column above CIR and SWIR suggests that microbially mediated processes are largely driven by the availability of hydrothermal vent-derived substrates.

The GeoChip results revealed various genes involved in organic matter oxidation with a high proportion for metabolism of aromatics. In hydrothermal vent fields, polycyclic aromatic hydrocarbons (PAHs) originate through a pyrolytic process (59) and are recalcitrant. Hydrothermally influenced water above CIR had a larger proportion of PAH-degrading genes (Fig. S2) compared to those at SWIR. Microbial communities in the deep ocean could use aromatic compounds as sources of carbon and energy (60). Bacterial genera capable of PAH-degradation have previously reported from SWIR (60). Genes encoding for hydrocarbon degradation were detected in the present study (Fig. S2), indicating a high potential for *in situ* bioremediation of hazardous petroleum compounds. In marine environments, nutrients too play a vital role in bioremediation of PAHs, wherein growth and cellular metabolism ratio of C:N:P is highly crucial (61). Elevated inorganic nitrogen and phosphorus in the deeper water at CIR and SWIR (Table 1) could favor PAH degradation. About 1.9% of genes at CIR and SWIR encoded for metabolism of aromatic carboxylic acids (Fig. S2). This implies that production of aromatic carboxylic acids could be occurring during degradation of various organic compounds, and they could be subsequently used as an alternate energy source.

Little is known about bacterially mediated N-cycle dynamics in mid-oceanic ridge (MOR) systems. At the Juan de Fuca Ridge, denitrification has been found to be the dominant N-loss pathway in sulfidic hydrothermal vent fluids that discharge from the subsurface (62). Of the two N-elimination pathways, i.e., denitrification and anammox, we found genes for nitrate, nitrite, and nitrous oxide reduction to be far more abundant throughout the water column. This observation provides genetic evidence on the predominance of the reductive phase of the N-cycle at CIR and SWIR (Fig. S3). Though nitrate accumulation was observed in the deeper water column (Table 1), we observed a marginal increase of the nutrient in turbid waters at CIR compared to near-bottom water. Hydrothermal vent fluids are nitrate replete compared to background deep-sea water (63). Although the UCDW is a major contributor of nutrients in the region (Fig. 3), nitrate regeneration either by nitrite re-oxidation, partial nitrification of hydrothermally derived ammonium, and/or di-nitrogen fixation and remineralization/nitrification of the newly fixed nitrogen could lead to additional nitrate input in the turbid water (63).

In the present study, genes for sulfur oxidation and sulfite reduction were the most dominant at CIR and SWIR (Fig. S4). In hydrothermal systems, sulfur-oxidizing bacteria are associated with the enrichment of genes mediating the denitrification pathway (8), suggesting coupling of S-cycle with other elemental pathways. During the final step of dissimilatory sulfate reduction, sulfite is reduced to form sulfide. Sulfite-reducing DsrAB-containing lineages possess the ability to oxidize hydrogen and short-chain fatty acids. They also possess three different carbon fixation mechanisms, viz., the CBB cycle, rTCA cycle, and the Wood–Ljungdahl pathway (64). In metagenomes from SWIR, potential genes encoding hydrogenases have been found to share high levels of similarity with sulfur- or sulfite-reducing bacteria such *Beggiatoa* sp. and *Desulfurobacterium thermolithotrophum* (8). These observations re-affirm that S-cycling at CIR and SWIR could be intrinsically linked to other elemental pathways. So far, little is also known about utilization of organosulfur compounds in deep-sea hydrothermal environments. We found genes for the secondary metabolite DMSP at all depths sampled in both regions, albeit at a lower abundance. Our findings are in congruence with those from hydrothermal sediments, where genetic potential for DMSP degradation has been observed to be very low and derived from biosynthesis rather than sinking particles (65). Demethylation of DMSP could be a less important process in hydrothermal systems.

Disruption in phosphate content can cause microbial cells to become susceptible to toxicity from transition metals (66). High concentration of metal ions has been shown to stimulate polyP hydrolysis and an efflux of metal-phosphate complexes in metal-resistant bacterium *Acidithiobacillus ferrooxidans* (67). Most of the genes for P-cycling in the present study were for polyP degradation (Fig. S5). At CIR and SWIR, an abundance of various metal-resistance genes was observed in turbid waters (Fig. S6) especially those showing resistance to arsenic, tellurium, and mercury. A larger proportion of polyP-degrading genes was also recorded in the turbid and near-bottom waters at CIR. Polyphosphate-metal ion interactions in turbid waters could help in reducing oxidative stress in many microorganisms (68) and enable them to tolerate higher concentrations of metals ions. Deep-ocean seafloor volcanic activities emit fluids rich in heavy metals and metalloids (69). These extreme environments are known to be inhabited by specialized microorganisms such as metal-oxidizing, metalloid-oxide-resistant, and metalloid-oxide-reducing bacteria. They occur in high numbers in ambient seawater around hydrothermal vents, bacterial films, and sulfide-rich rocks (70). Due to their high metal-resistance and metal-transformation properties, isolated bacterial strains have been suggested to have applications in bioremediation (71, 72) and applied biometallurgy (70). Lead-arsenic-rich microbial filamentous net-like clusters have been observed in arsenic- and lead-enriched high-temperature black smoker from the Manus back-arc basin, Papua New Guinea. Therefore, higher abundance of metal-resistant genes especially in turbid waters suggests the occurrence of hydrothermal vents in the vicinity of our sampling site. Moreover, a new ultramafic-hosted hydrothermal field has been reported (12) near the sampling location CTD 17, in southern Central Indian Ridge.

In this study, signal intensities of genes from dominant genera in turbid waters were for multiple functions mainly C-fixation via CBB pathway, C-degradation, detoxification of arsenic, chromium, mercury, tellurium, remediation of aromatics, polycyclic aromatics, sulfite reduction, sulfide oxidation, and polyphosphate degradation. Hence, there is a need of more extensive studies to understand their distribution and ecology in deep‐sea hydrothermal environments. Our results also reveal enhanced functional gene diversity in turbid waters (Table 1). However, it was observed that microbial communities in deeper waters were largely impacted by the availability of inorganic nutrients (Fig. 5) transported by the UCDW, highlighting its importance in sustaining and structuring chemoautotrophic microbial communities along CIR and SWIR.

In sum, this study provides deeper insights into depth-driven differences of microbial functional genes and their diversity along CIR and SWIR. GeoChip analysis revealed the predominance of C-metabolism over other key functional gene categories in the microarray. Localized hydrothermal activity increased the diversity of microbial functional genes moreso at SWIR. Generally, an elevated abundance of chemoautototrophs capable of mediating multiple biogeochemical processes, such as the degradation of aromatic compounds, metal resistance, and sulfide oxidation, was observed in turbid waters from both regions possibly due hydrothermal circulation. These findings indicate that there is a great potential for harnessing novel microbes from deep-sea ecosystems for ecological remediation and restoration through the degradation of recalcitrant environmental pollutants and mining waste products. This study also highlights that the intrusion of nutrient-rich UCDW has a fundamentally larger influence on microbial metabolic potential and functional gene diversity at >2,000 m in both regions. This could affect biological productivity in the deep sea. Recently, it has been suggested that Rodriguez Triple Junction (RTJ) is not a dispersal barrier for larvae of many vent endemic animals (43). However, findings in this study indicate that the RTJ is still a barrier for cross-ridge dispersal of vent endemic species as supported by the spatial extent of water masses. Future studies directed at understanding biogeography of microorganisms along MORs could consider inclusion of oceanographic features to draw stronger inferences on variability of microbial communities, larval dispersal mechanisms, and genetic connectivity.

## Data Availability

The GeoChip 5.0S data have been deposited in NCBI Gene Expression Omnibus (GEO, https://www.ncbi.nlm.nih.gov/geo/) under the accession number 22156631.

## SUPPLEMENTAL MATERIAL

Supplemental material spectrum.03306-23-s0001.pdf Fig. S1 to S8.

ASM does not own the copyrights to Supplemental Material that may be linked to, or accessed through, an article. The authors have granted ASM a non-exclusive, world-wide license to publish the Supplemental Material files. Please contact the corresponding author directly for reuse.
