# Supplementary material for: Upper circumpolar deep water influences microbial functional gene composition and diversity along the southern Central Indian Ridge and eastern Southwest Indian Ridge
Source: Microbiol Spectr. 2024 Dec 27;13(2):e03306-23. doi: 10.1128/spectrum.03306-23 (PMC11792553; doi:10.1128/spectrum.03306-23)
Supplement: Supplemental material — Fig. S1 to S8. [file spectrum.03306-23-s0001.pdf]

Supplemental Material

FIG S1 Proportion in percentage of relative abundance of genes ± standard deviation for (a) C-fixation (b) C-degradation and (c) methane metabolism at CIR and SWIR.

FIG S2 Proportion in percentage of relative abundance of genes ± standard deviation for organic remediation at CIR and SWIR.

FIG S3 Proportion in percentage of relative abundance of genes ± standard deviation mediating various N-cycle processes at CIR and SWIR.

FIG S4 Proportion in percentage of genes ± standard deviation involved in sulphur cycling at CIR and SWIR.

FIG S5 Proportion in percentage of genes ± standard deviation for phosphorus cycling at CIR and SWIR.

FIG S6 Proportion in percentage of genes ± standard deviation responsible for metal resistance at CIR and SWIR.

FIG S7 Correlation plot between environmental variables and diversity indices at CIR. The colour of the dots denotes the sign of the correlation (highly positive in dark blue to highly negative in red). Only significant values ($P < 0.05$) have been coloured. Chl. a, chlorophyll a; DMn, dissolved manganese; DO, dissolved oxygen, pH, hydrogen ion concentration and TPC, total prokaryotic cells.

FIG S8 Correlation plot between environmental variables and diversity indices at SWIR.

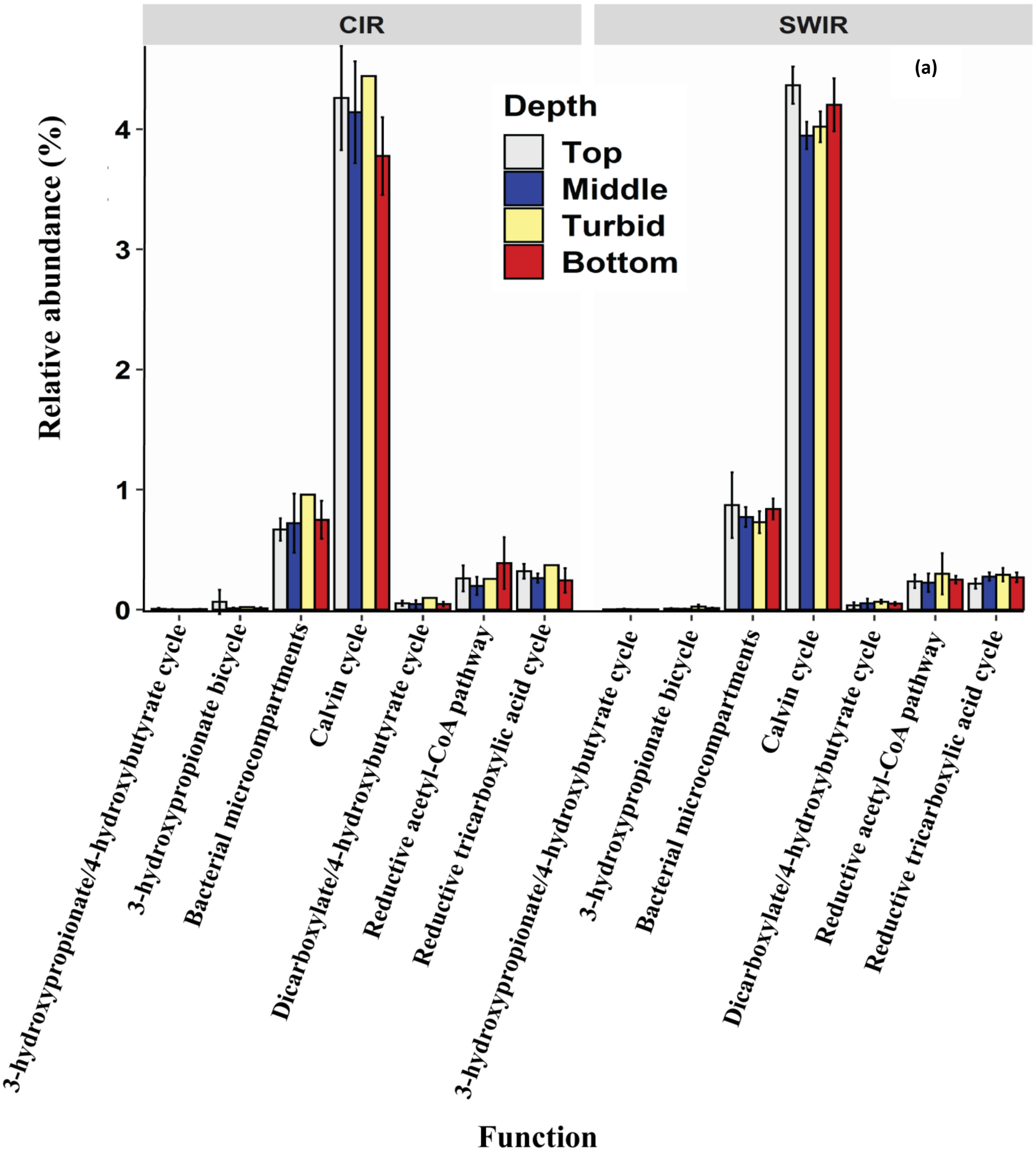
CIR
SWIR
(a)
Depth
Top
Middle
Turbid
Bottom
Relative abundance (%)
0
1
2
3
4
3-hydroxypropionate/4-hydroxybutyrate cycle
3-hydroxypropionate bicycle
Bacterial microcompartments
Calvin cycle
Dicarboxylate/4-hydroxybutyrate cycle
Reductive acetyl-CoA pathway
Reductive tricarboxylic acid cycle
Function

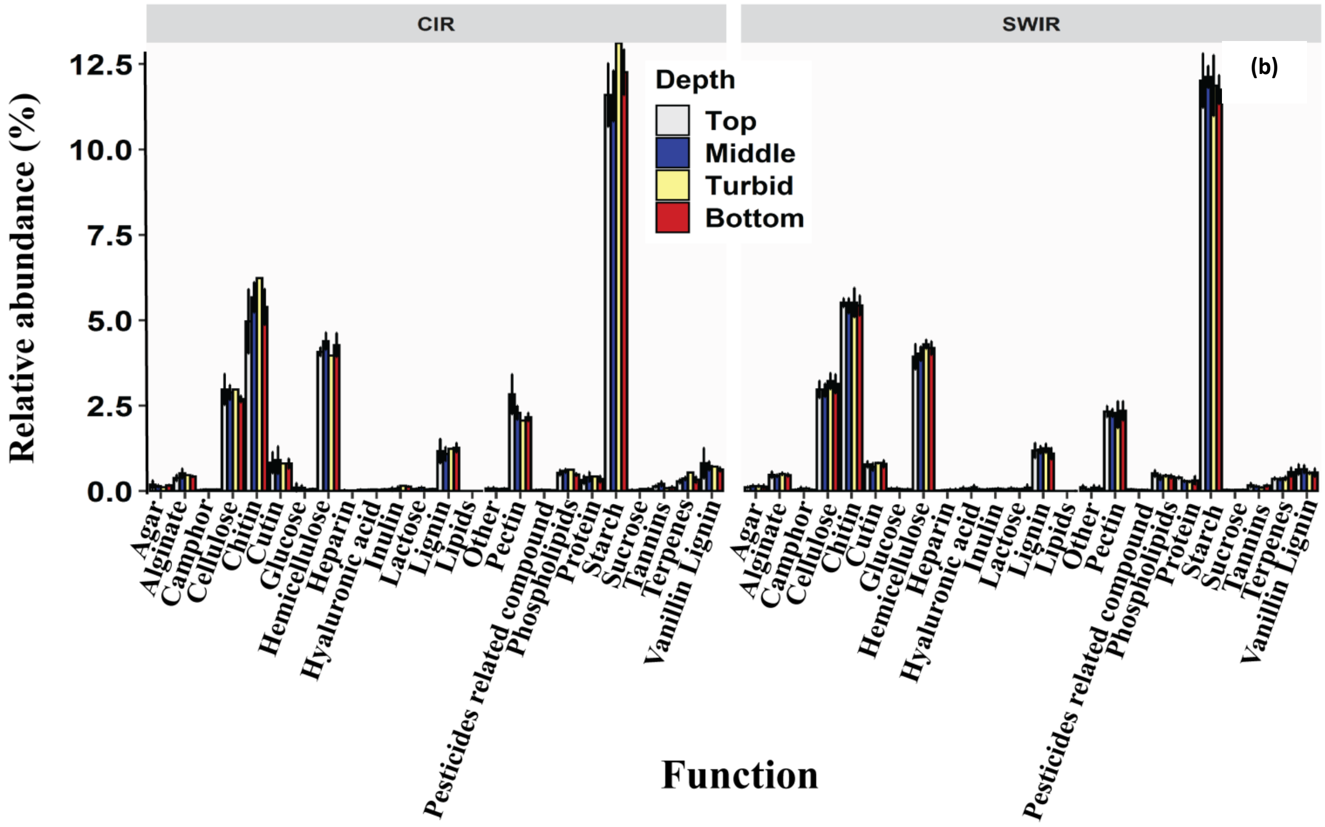

CIR
SWIR
(b)
Relative abundance (%)
12.5
10.0
7.5
5.0
2.5
0.0
Depth
Top
Middle
Turbid
Bottom
Agar
Alginate
Camphor
Cellulose
Chitin
Cutin
Glucose
Hemicellulose
Heparin
Hyaluronic acid
Inulin
Lactose
Lignin
Lipids
Other
Pectin
Pesticides related compound
Phospholipids
Protein
Starch
Sucrose
Tannins
Terpenes
Vanillin Lignin
Function

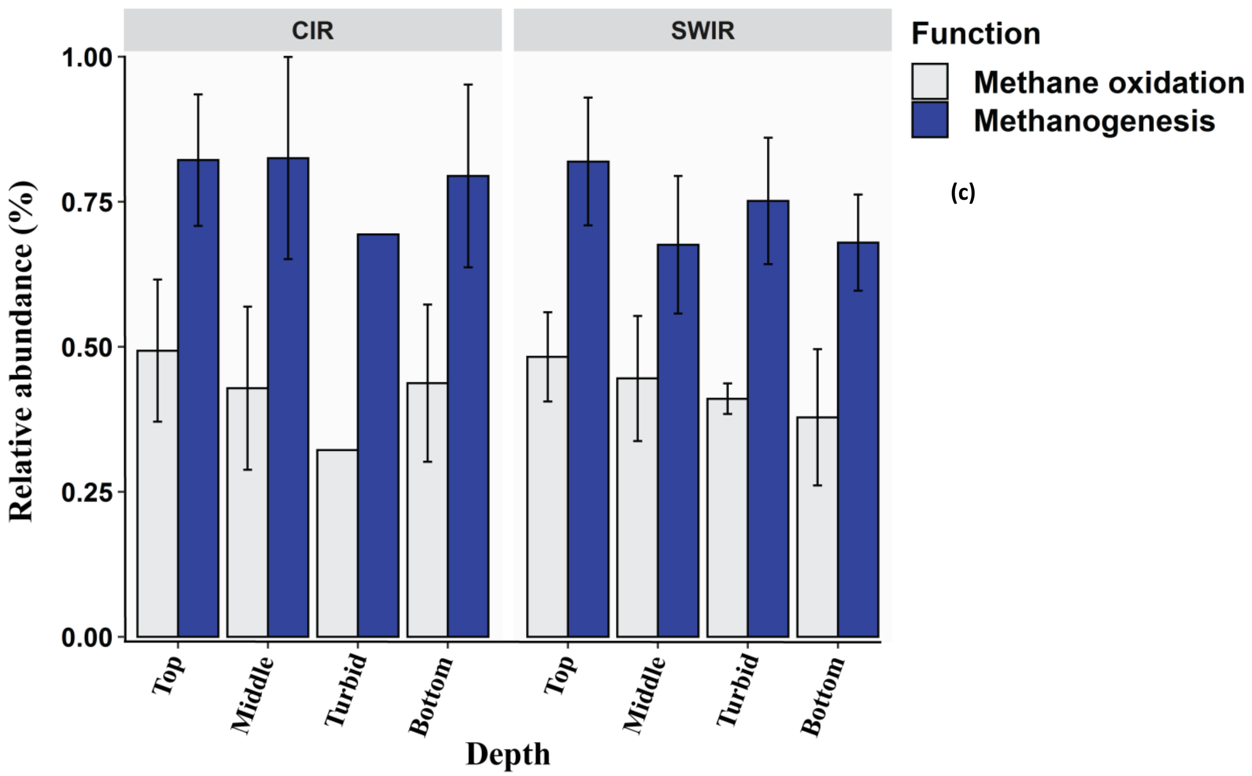
CIR
SWIR
Function
Methane oxidation
Methanogenesis
(c)
1.00
0.75
0.50
0.25
0.00
Relative abundance (%)
Top
Middle
Turbid
Bottom
Top
Middle
Turbid
Bottom
Depth

FIG S1

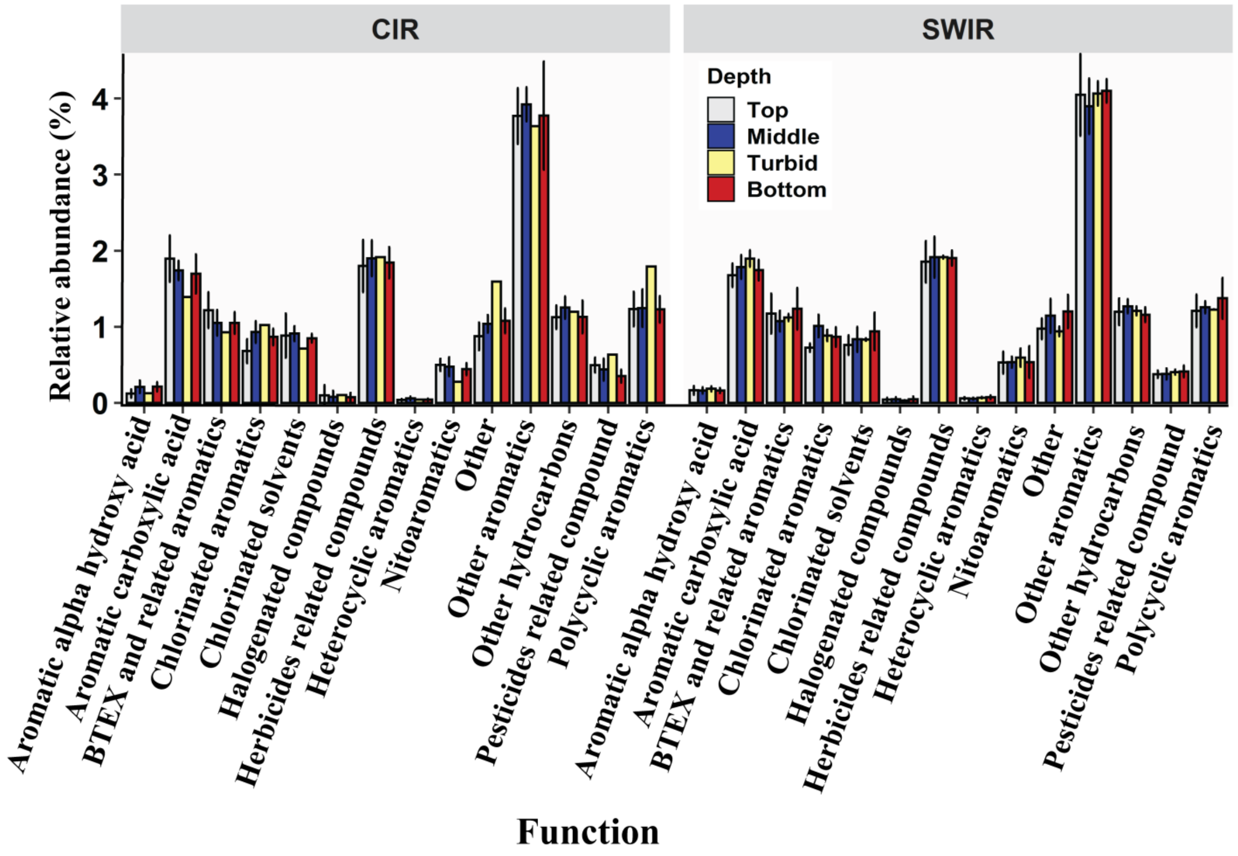
CIR
SWIR
Depth
Top
Middle
Turbid
Bottom
Relative abundance (%)
0
1
2
3
4
Aromatic alpha hydroxy acid
Aromatic carboxylic acid
BTEX and related aromatics
Chlorinated aromatics
Chlorinated solvents
Halogenated compounds
Herbicides related compounds
Heterocyclic aromatics
Nitoaromatics
Other
Other aromatics
Other hydrocarbons
Pesticides related compound
Polycyclic aromatics
Function

FIG S2

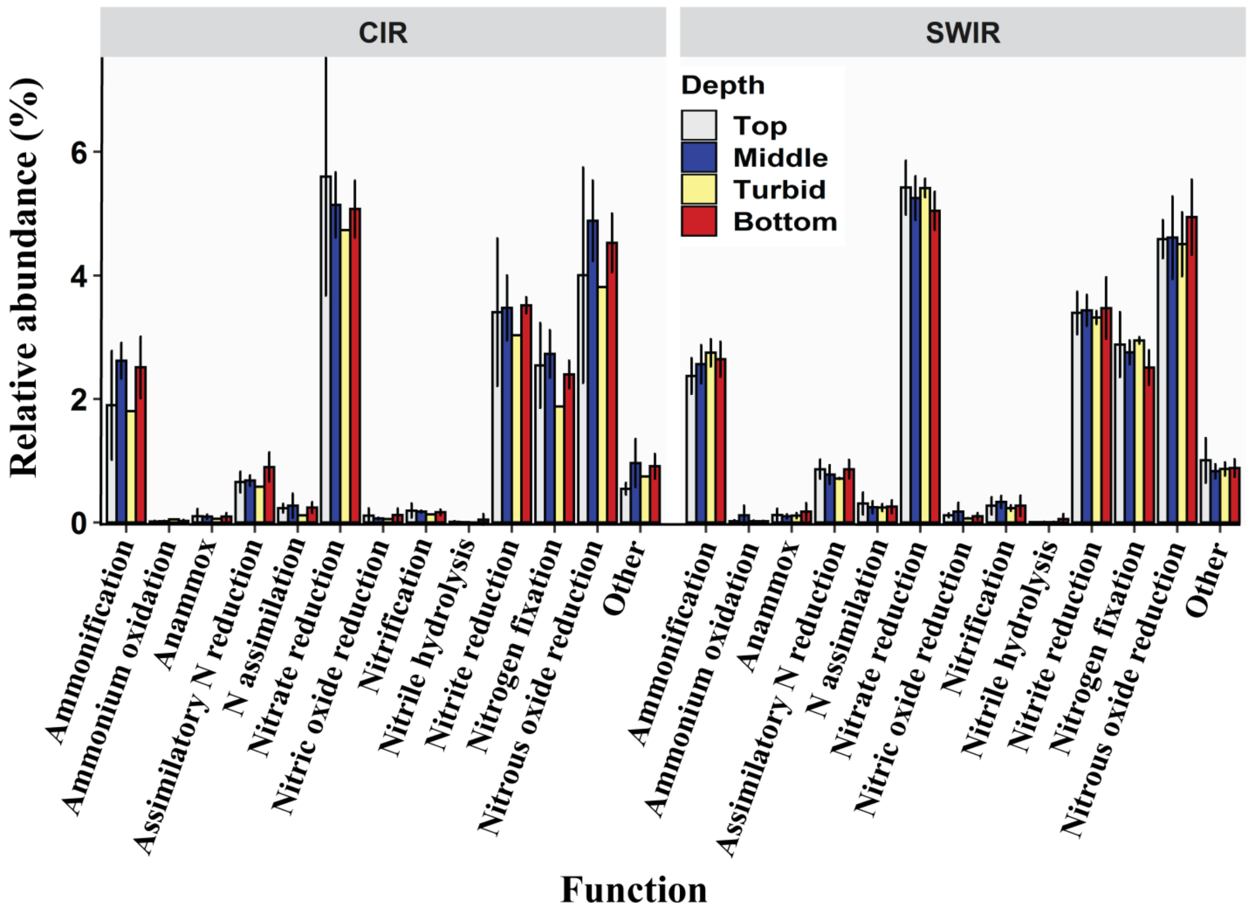
CIR
SWIR
Depth
Top
Middle
Turbid
Bottom
Relative abundance (%)
0
2
4
6
Ammonification
Ammonium oxidation
Anammox
Assimilatory N reduction
N assimilation
Nitrate reduction
Nitric oxide reduction
Nitrification
Nitrile hydrolysis
Nitrite reduction
Nitrogen fixation
Nitrous oxide reduction
Other
Function

FIG S3

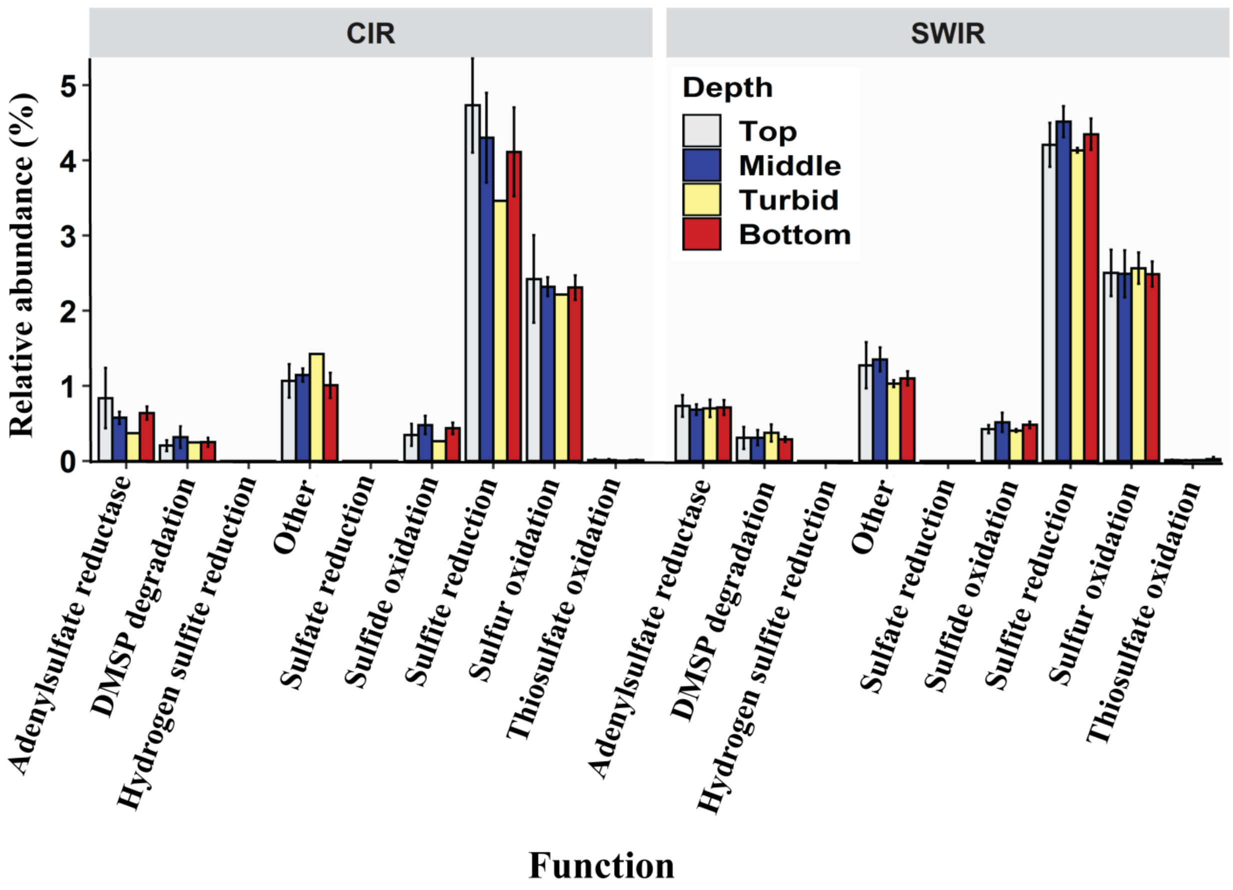
CIR
SWIR
Depth
Top
Middle
Turbid
Bottom
Relative abundance (%)
0
1
2
3
4
5
Adenylsulfate reductase
DMSP degradation
Hydrogen sulfite reduction
Other
Sulfate reduction
Sulfide oxidation
Sulfite reduction
Sulfur oxidation
Thiosulfate oxidation
Function

FIG S4

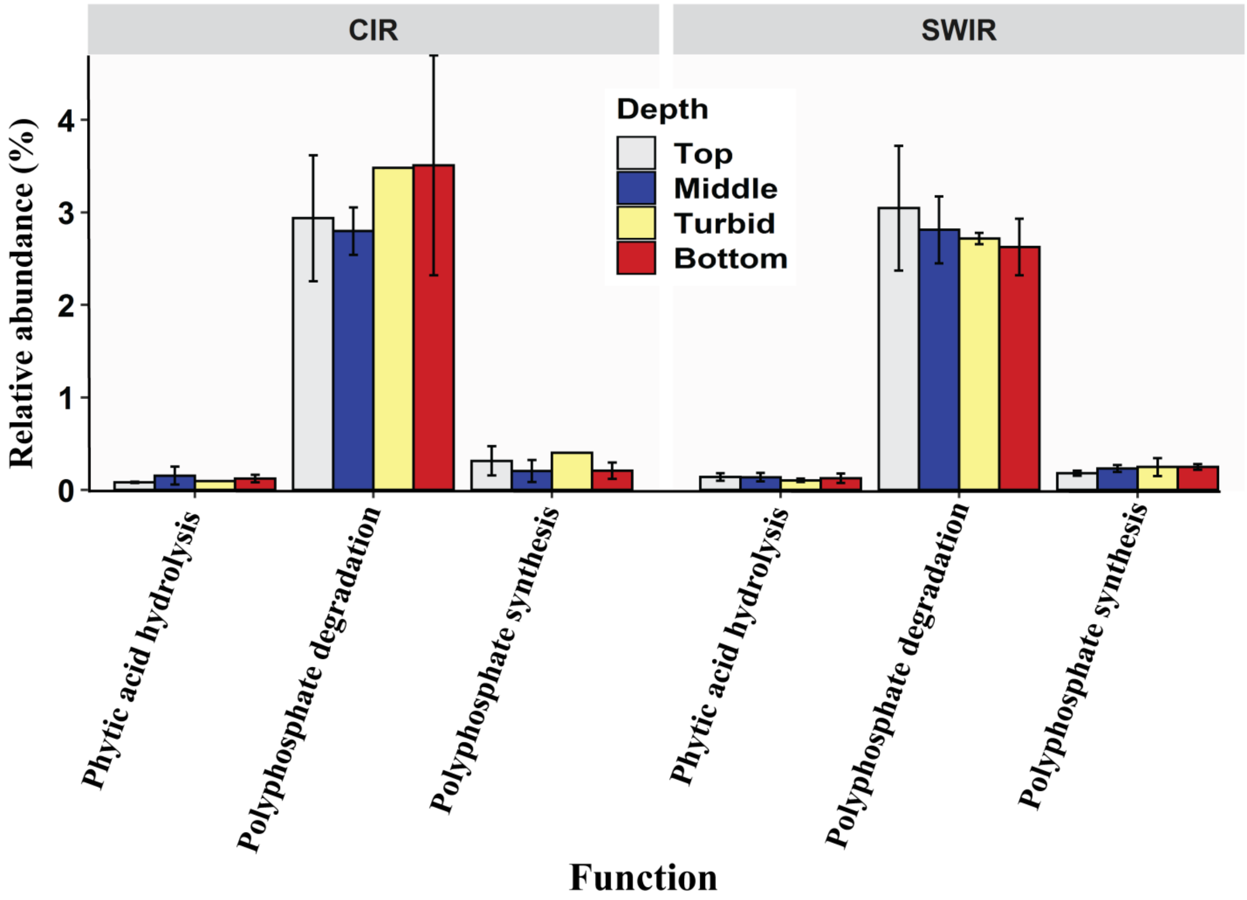
CIR
SWIR
Depth
Top
Middle
Turbid
Bottom
Relative abundance (%)
0
1
2
3
4
Phytic acid hydrolysis
Polyphosphate degradation
Polyphosphate synthesis
Phytic acid hydrolysis
Polyphosphate degradation
Polyphosphate synthesis
Function

FIG S5

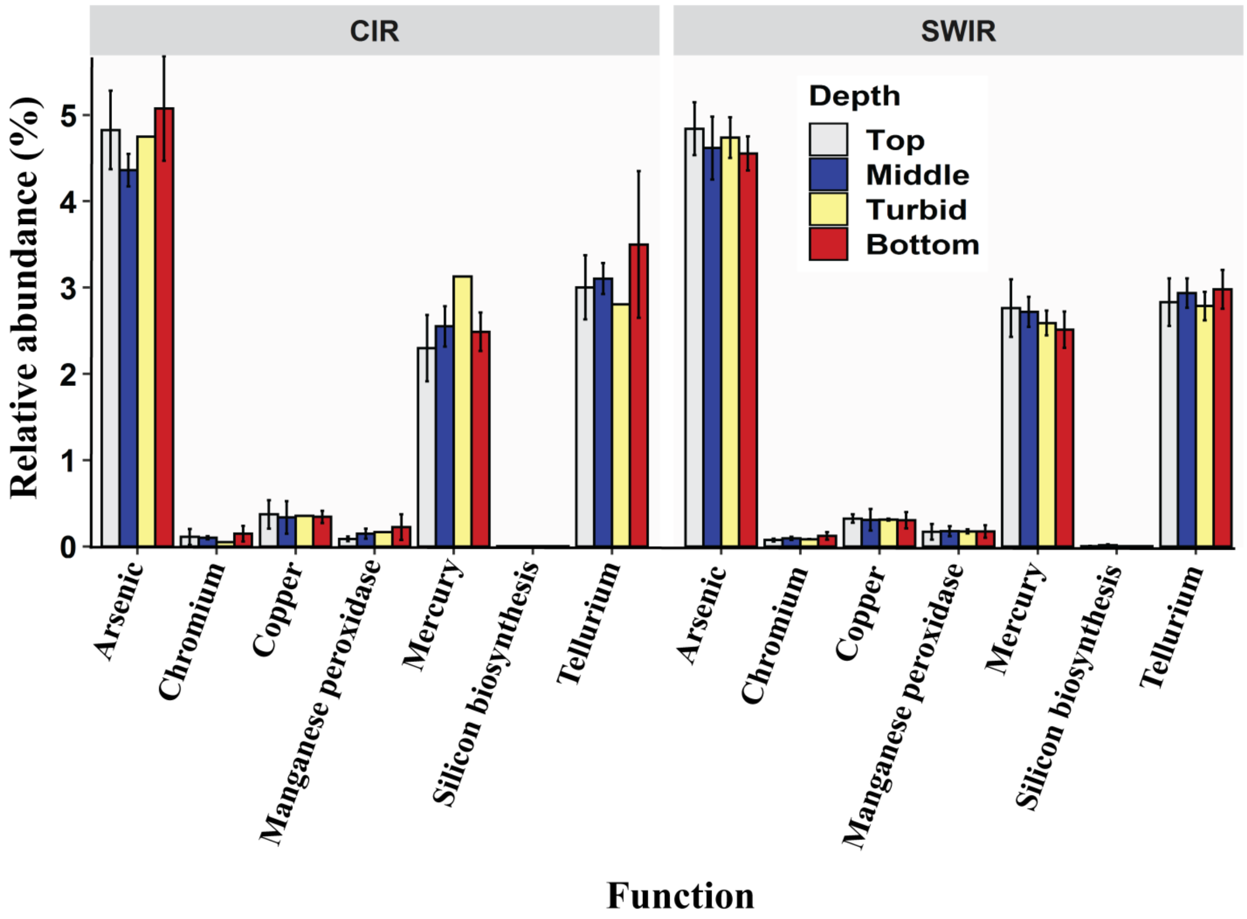

CIR
SWIR
Depth
Top
Middle
Turbid
Bottom
Relative abundance (%)
0
1
2
3
4
5
Arsenic
Chromium
Copper
Manganese peroxidase
Mercury
Silicon biosynthesis
Tellurium
Function

FIG S6

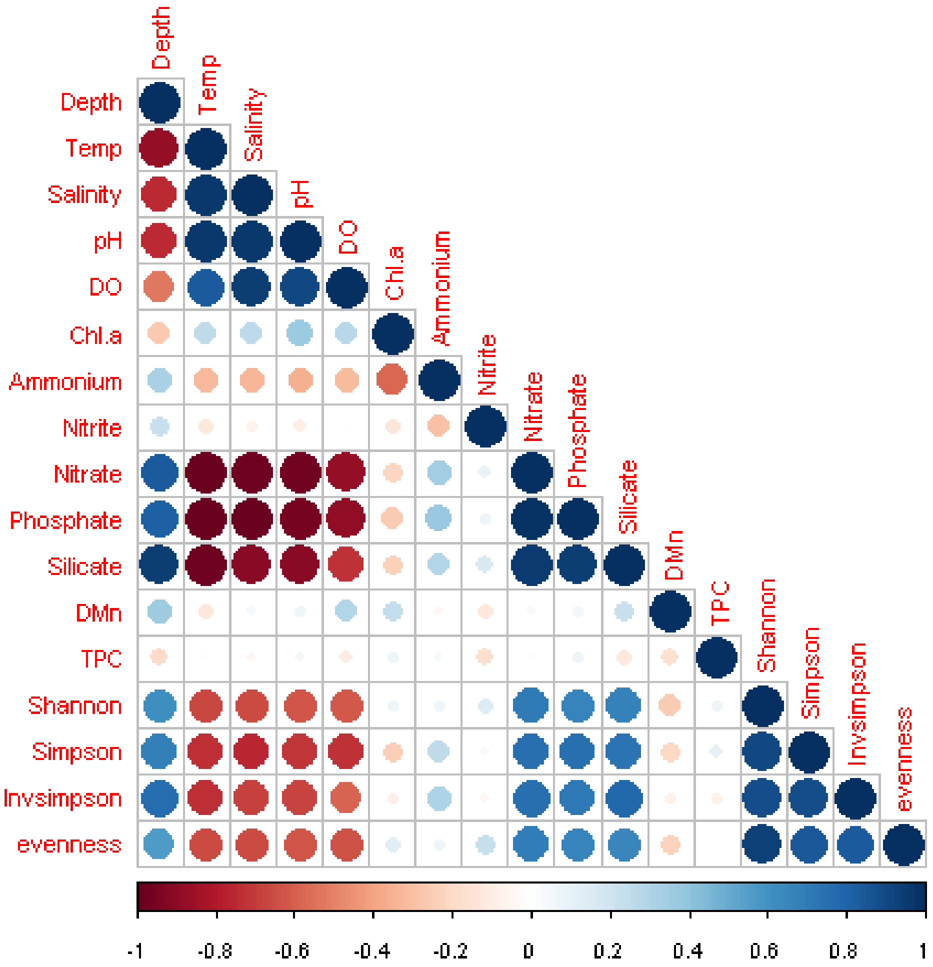
Depth
Temp
Salinity
pH
DO
Chl.a
Ammonium
Nitrite
Nitrate
Phosphate
Silicate
DMn
TPC
Shannon
Simpson
Invsimpson
evenness
-1
-0.8
-0.6
-0.4
-0.2
0
0.2
0.4
0.6
0.8
1

FIG S7

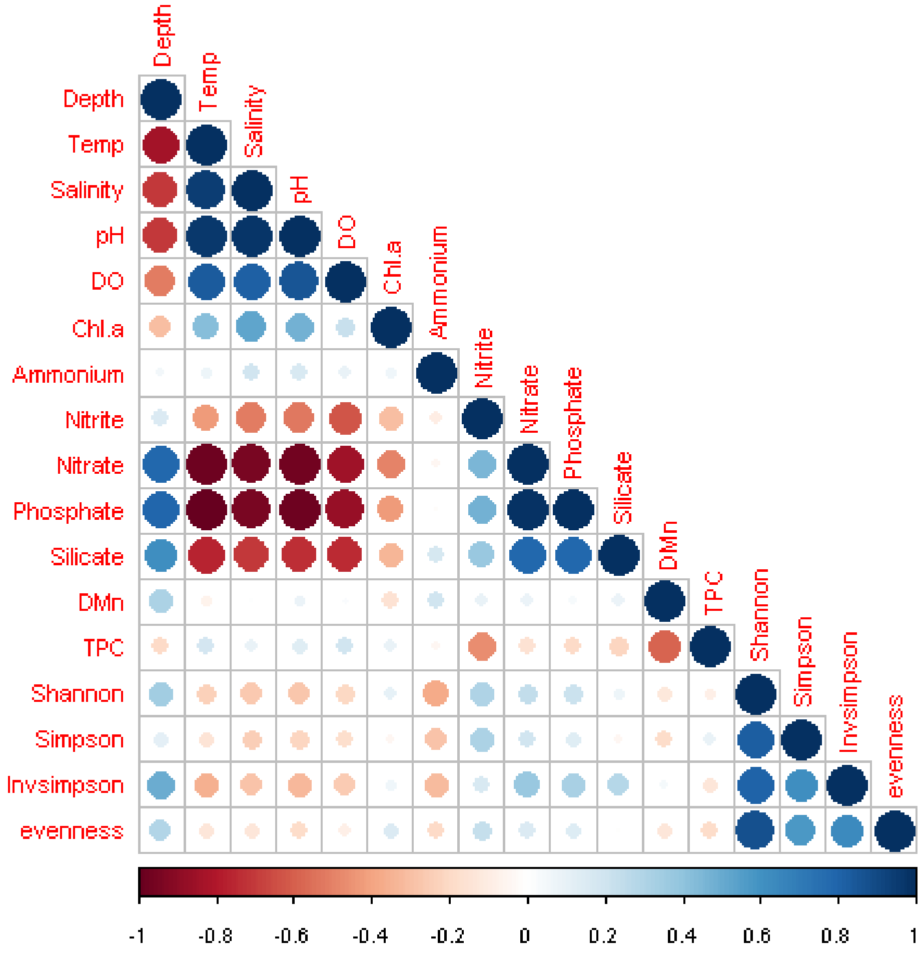
Depth
Temp
Salinity
pH
DO
Chl.a
Ammonium
Nitrite
Nitrate
Phosphate
Silicate
DMn
TPC
Shannon
Simpson
Invsimpson
evenness
-1
-0.8
-0.6
-0.4
-0.2
0
0.2
0.4
0.6
0.8
1

FIG S8
